# Impact of Noise on a Dynamical System: Prediction and Uncertainties from a Swarm-Optimized Neural Network

**DOI:** 10.1155/2015/145874

**Published:** 2015-07-30

**Authors:** C. H. López-Caraballo, J. A. Lazzús, I. Salfate, P. Rojas, M. Rivera, L. Palma-Chilla

**Affiliations:** Departamento de Física y Astronomía, Universidad de La Serena, Casilla 554, La Serena, Chile

## Abstract

An artificial neural network (ANN) based on particle swarm optimization (PSO) was developed for the time series prediction. The hybrid ANN+PSO algorithm was applied on Mackey-Glass chaotic time series in the short-term *x*(*t* + 6). The performance prediction was evaluated and compared with other studies available in the literature. Also, we presented properties of the dynamical system via the study of chaotic behaviour obtained from the predicted time series. Next, the hybrid ANN+PSO algorithm was complemented with a Gaussian stochastic procedure (called *stochastic* hybrid ANN+PSO) in order to obtain a new estimator of the predictions, which also allowed us to compute the uncertainties of predictions for noisy Mackey-Glass chaotic time series. Thus, we studied the impact of noise for several cases with a white noise level (*σ*_*N*_) from 0.01 to 0.1.

## 1. Introduction

Currently, the prediction of time series has played an important role in many science fields of practical application as engineering, biology, physics, meteorology, and so forth. In particular, and due to their dynamical properties, the analysis and prediction of chaotic time series have been of interest for the science community. In general, the chaotic time series are usually modeled by delay-differential equations; standard examples are the Mackey-Glass system [1], or the Ikeda equation [2] (for more examples, see [3]). Also, many methods have been used in the chaotic time series analysis [4]. However, in the last decades, different types of artificial neural networks (ANN) have been widely used for forecasting of chaotic time series, for example, backpropagation algorithm [5], radial basic function [6], and recurrent network [7].

On the other hand, the analysis of real-life time series requires taking into account the error propagation of input uncertainties. The observed data could be contaminated for different instrumental noise types as white noise or proportional to signal (the latter mainly arises from instrumental calibration). In the modeling of chaotic time series, the impact of noise can be treated as errors-invariable problem where the noise is propagated into the prediction model. In the literature, the noisy impact on chaotic time series prediction has been barely considered. We can find studies where the algorithms were tested from a theoretical point of view (e.g., see [8–12]) and works where the implementation was applied on real-life time series (e.g., see [9, 13, 14]). In addition, some authors have proposed a modification to the standard methods in order to improve the performance prediction in presence of noise [9, 14].

In this work, we used the Mackey-Glass chaotic time series in order to study the short-term prediction (*x*(*t* + 6)) with an artificial neural network optimized with a particle swarm algorithm (ANN+PSO). The method was applied on noiseless and noisy chaotic time series. In order to carry out the error propagation of the input noise, this hybrid algorithm was complemented with a Gaussian stochastic procedure to compute a new estimator of the predictions and their uncertainties. Note that ANNs have been used in combination with PSO in several applications. Principally, these applications include feed-forward neural network training [15–18], design of recurrent neural networks [19], design of radial basis function networks [20], and neural network control for nonlinear processes [21]. In addition, there are several current versions of PSO available in the literature (e.g., see the following reviews [22–24]), but our application uses a standard PSO with inertial weight [25]. In this point, the use of a PSO with inertial weight is based on the following reasons: (1) this version of PSO is easy to understand and implement due to its simple concept and learning strategy; (2) as pointed out in [26], the PSO with inertia weight [25] and PSO with constriction factor [27] are mathematically equivalent, and PSO with constriction factor can be considered as a special case of PSO with inertia weight [22, 26] (note that this equivalence can be applied to other improved PSO algorithms that include a varying inertia weight schedule); (3) inertia weight PSO algorithm is quite stable to population changes [23]; (4) the advantages and disadvantages of variants of PSO depend on the problem to solve [22–24]; (5) as a first approach for study of noise effect on dynamical systems using an ANN combined with inertia weight PSO algorithm, the present study may motivate and help the researchers working in the field of evolutionary algorithms to develop new hybrid models or to apply other existing PSO models to solve this problem. To the best of the authors' knowledge, there is no application for forecasting the noisy chaotic time series such as the one presented here, using a hybrid method that combined ANN with PSO algorithm.

Organization of this paper is as follows. In Section 2, we present a detailed description of the hybrid ANN+PSO method. Sections 3 and 4 present the simulation, algorithm implementation, and the principal results obtained for the forecasting of noiseless chaotic time series and noisy time series, respectively. Finally, conclusions are given in Section 5.

## 2. Hybrid ANN+PSO Algorithm

Artificial neural networks (ANNs) are similar to biological neural networks in performing functions collectively and in parallel using connection nodes. Thus, ANNs are a family of statistical learning algorithms biologically inspired.

In this study, we consider one of the most successful and frequently used types of neural networks: a multilayer feed-forward neural network with a backpropagation learning algorithm (gradient descent error). This ANN was implemented replacing standard backpropagation with particle swarm optimization (PSO).

PSO is a population-based optimization tool, where the system is initialized with a population of random particles and the algorithm searches for optima by updating generations [28]. In each iteration, the velocity of each particle *j* is calculated according to the following formula [29]:

(1) $$\begin{array}{c} v_{j}^{k+1}=\omega v_{j}^{k}+c_{1}r_{1}\left(\psi_{j}^{k}-s_{j}^{k}\right)+c_{2}r_{2}\left(\psi_{g}^{k}-s_{j}^{k}\right), \end{array}$$

where *s* and *v* denote a particle position and its corresponding velocity in a search space, respectively. *k* is the current step number, *ω* is the inertia weight, *c*_1_ and *c*_2_ are the acceleration constants, and *r*_1_, *r*_2_ are elements from two random sequences in the range (0,1). *s*_*j*_^*k*^ is the current position of the particle, *ψ*_*j*_^*k*^ is the best one of the solutions that this particle has reached, and *ψ*_*g*_ is the best solutions that all the particles have reached. In general, the value of each component in *v* can be clamped to the range [−*v*_max_, +*v*_max_] control excessive roaming of particles outside the search space [28, 29]. After calculating the velocity, the new position of each particle is

(2) $$\begin{array}{c} s_{j}^{k+1}=s_{j}^{k}+v_{j}^{k+1}. \end{array}$$

The procedure to calculate the output values, using the input values, is described in detail in [30].

The net inputs (*N*) are calculated for the hidden neurons coming from the inputs neurons. In the case of a neuron in the hidden layer, one has

(3) $$\begin{array}{c} N_{i}^{h}=\overset{n}{\underset{i}{\sum }}w_{i,j}^{h}p_{i}+b_{i,j}^{h}, \end{array}$$

where *p*_*i*_ is the vector of the inputs of the training, *w*_*i*,*j*_^*h*^ is the weight of the connection among the input neurons with the hidden layer *h*, and the term *b*_*i*,*j*_^*h*^ corresponds to the bias of the neuron of the hidden layer *h*, reached in its activation. The PSO algorithm is very different than any of the traditional methods of training [28]. Each neuron contains a position and velocity. The position corresponds to the weight of a neuron (*s*_*i*_^*k*^ → *w*_*i*,*j*_^*h*^). The velocity is used to update the weight (*v*_*i*_^*k*+1^ → *w*_*i*,*j*_′). Starting from these inputs, the outputs (*y*_*i*_) of the hidden neurons are calculated, using a transfer function *f* ^*h*^ associated with the neurons of this layer:

(4) $$\begin{array}{c} y_{i}=f^{h}\left(\overset{n}{\underset{i}{\sum }}w_{i,j}^{h}p_{i}+b_{i,j}^{h}\right). \end{array}$$

The transfer functions *f* ^*h*^ can be linear or nonlinear. We used one hidden layer with *f*_*i*_^*h*^ as a tangent hyperbolic function (*tansing*) and *f*_*j*_^*h*^ as a linear function in the output layer:

(5) $$\begin{array}{c} f\left(N_{i}\right)=\frac{e^{N_{i}}-e^{-N_{i}}}{e^{N_{i}}+e^{-N_{i}}}. \end{array}$$

All the neurons of the ANN have an associated activation value for a given input pattern, and the algorithm continues finding the error that is presented for each neuron, except those of the input layer. After finding the output values, the weights of all layers of the network are actualized *w*_*i*,*j*_ → *w*_*i*,*j*_′ by PSO, using (1) and (2) [29]. The velocity is used to control how much the position is updated. On each step, PSO compares each weight using the data set. The network with the highest fitness is considered the global best. The other weights are updated based on the global best network rather than their personal error or fitness [28]. In this paper, we used the mean square error (MSE) to determine network fitness for the entire training set:

(6) $$\begin{array}{c} MSE=\frac{\sum_{i=1}^{n}{\left(Y_{i}^{true}-Y_{i}^{calc}\right)}^{2}}{n}, \end{array}$$

where *Y*_*i*_^true^ is the real data and *Y*_*i*_^calc^ is the calculated output value obtained from the normalized output (*y*_*i*_) of the network. This process was repeated for the total number of patterns in the training set. For a successful process, the objective of the algorithm is to modernize all the weights minimizing the total root mean squared error (RMSE):

(7) $$\begin{array}{c} RMSE=\sqrt{MSE}, \end{array}$$

(8) $$\begin{array}{c} \varepsilon =\min\left(RMSE\right). \end{array}$$

**Figure 1:**
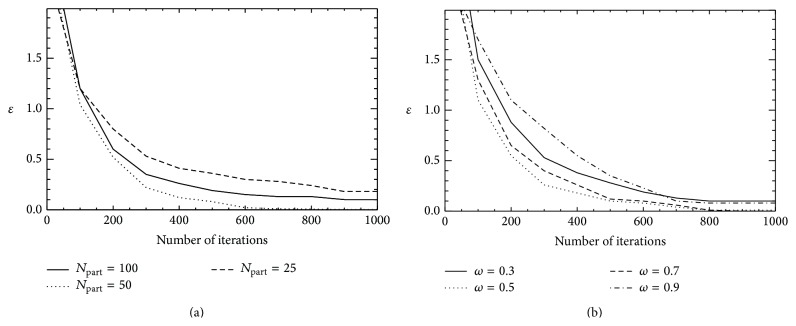
Illustration of the behaviour of some parameters of the ANN+PSO against the number of iterations. (a) and (b) correspond to the number of particles in the swarm (*N*_part_) and the inertia weight (*ω*), respectively.

In PSO, the inertial weight *ω*, the constants *c*_1_ and *c*_2_, the number of particles *N*_part_, and the maximum speed of particle summarize the parameters to synchronize for their application in a given problem. Then, an exhaustive trial-and-error procedure was applied to tune the PSO+ANN parameters. Firstly, the effect of population *N*_part_ is analyzed for values of 25 to 100 individuals in the swarm. For other applications, some authors have shown that a larger swarm increases the number of function evaluations to converge to an error limit [31]. In addition, Shi and Eberhart [32] illustrated that the population size has hardly any effect on the performance of a swarm algorithm.  Figure 1(a) shows that the best population to solve the problem is of 50 individuals. Next, the effect of *ω* is analyzed for values of 0.1 to 0.9.  Figure 1(b) shows the values of *ω* that favoured the search of the particles and accelerated the convergence. This figure shows that for a linearly decreasing inertia weight starting at 0.7 and ending at 0.5, the PSO+ANN presents a good convergence. In other aspect, a usual choice for the acceleration coefficients *c*_1_ and *c*_2_ is *c*_1_ = *c*_2_ [31]. The effect of variation of constants was evaluated for the commonly used values of *c*_1_ and *c*_2_ such as 1.49 and 2.00 [31, 32]. For this analysis, *c*_1_ = *c*_2_ = 1.49 presents a better convergence than other values.  Table 1 shows the selected parameters for this hybrid algorithm.

The step-to-step approach of PSO+ANN can be summarized as follows.

**Step 1 .**

Initialize the positions (weights and biases) and velocities of a group of particles randomly. The particles represent the weight vectors of ANN, including biases. The dimension of the search space is therefore the total number of weights and biases.

**Step 2 .**

The ANN is trained using the initial particles position in PSO. The learning error produced from ANN network can be treated as particles fitness value according to initial weight and bias. The current best fitness achieved by particle *j* is set as *ψ*_*j*_^*k*^. The *ψ*_*j*_^*k*^ with best value is set as *ψ*_*g*_ and this value is stored.

**Step 3 .**

Evaluate the desired optimization fitness function (7) over a given data set.

**Step 4 .**

Compare the evaluated fitness value of each particle (*F*_*j*_) with its value. If *F*_*j*_ < *ψ*_*j*_^*k*^, then *ψ*_*j*_^*k*^ = *s*_*j*_^*k*^ is the coordinates corresponding to best particle so far.

**Step 5 .**

The objective function value is calculated for new positions of each particle. If a better position is achieved by an agent, *ψ*_*j*_^*k*^ value is replaced by the current value. As in Step 1, *ψ*_*g*_ value is selected among *ψ*_*j*_^*k*^ values. If the new *ψ*_*g*_ value is better than the previous value, it is replaced by the current *ψ*_*g*_ value and this value is stored. If *F*_*j*_ < *ψ*_*g*_, then *ψ*_*g*_ = *s*_*j*_^*k*^ is the particle having the overall best fitness over all particles in the swarm.

**Step 6 .**

The learning error at current epoch will be reduced by changing the particles position, which will update the weight and bias of the network. Change the velocity and location of the particle according to movement equations (1) and (2). The new sets of positions (weights and biases) are produced by adding the calculated velocity value to the current position value. Then, the new sets of positions are used to produce new learning error in ANN.

**Step 7 .**

This process is repeated until the stopping conditions either minimum learning error or maximum number of iterations are met and then stop; otherwise, loop to Step 3 until convergence.

**Step 8 .**

The optimum weight and biases for ANN model are obtained by PSO. Best training process is obtained for ANN.

In our time series analysis, if the input noise level contribution is available, the RMSE in the training phase will be computed as follows:

(9) $$\begin{array}{c} RMSE=\sqrt{\frac{1}{n}\overset{n}{\underset{i=1}{\sum }}\frac{{\left(Y_{i}^{cal}-Y_{i}^{true}\right)}^{2}}{\sigma_{N,i}^{2}}}, \end{array}$$

where *σ*_*N*,*i*_ is the noise level of each *i*-element. Note that *σ*_*N*,*i*_ = *σ*_*N*_, for a white noise assumption.

Henceforth, we refer as* the standard ANN*+*PSO* to the hybrid ANN+PSO defined above.

### 2.1. The Stochastic ANN+PSO

Up to now, the standard ANN+PSO is not developed to carry out the error propagation of the input noise level contribution. Nevertheless, once the* standard* ANN+PSO has been executed and has provided the optimal topology, we can apply an additional method in order to compute uncertainty of the prediction.

Note that once the topology is established (number of hidden layers, neurons in each hidden layer, transfer functions *f* ^*h*^, and weights and biases (*w*_*i*,*j*_^*h*^ and *b*_*i*,*j*_^*h*^)), the neural network acts as a function (called* function ANN*) whose output only depends on the input vector (see (4)). The idea is to generate simulations from the input data (*d*_*i*_ ≡ *d*(*t*)) via Gaussian random number generator in order to propagate the intrinsic data noise through the* function ANN*.

For each *i*-element of the input time series, we generate *k*-simulations as

(10) $$\begin{array}{c} d_{i,k}=d_{i}+{GR}_{k}\left(\sigma_{N,i}\right), \end{array}$$

where the input noise level *σ*_*N*,*i*_ is known. GR(*σ*_*N*,*i*_) is a random number generator following a Gaussian distribution with mean zero and standard deviation equal to *σ*_*N*,*i*_^2^.

Finally, for the *i*th element, each *k* input data set *d*_*i*,*k*_ provides an output *y*_*i*,*k*_. These *y*_*i*,*k*_ are used in the estimation of a new estimator of prediction (${\hat{y}}_{i}$) and an error on the prediction ($\sigma_{\hat{y}}$) as follows:

(11) $$\begin{array}{c} {\hat{y}}_{i}=\langle y_{i,k}\rangle ,\\ \sigma_{\hat{y}}={\langle y_{i,k}^{2}\rangle }^{1/2}. \end{array}$$

## 3. Noiseless Chaotic Time Series Prediction

We computed the chaotic time series from the Mackey-Glass time-delay differential system [1, 33], which is described as follows:

(12) $$\begin{array}{c} \frac{dx}{dt}=\beta x\left(t\right)+\frac{\alpha x\left(t-\tau \right)}{1+x{\left(t-\tau \right)}^{10}}, \end{array}$$

where *x* (unitless) is the series in the time *t* and *τ* is the time delay. Here, we assumed that *α* = 0.2, *β* = 0.1, and *x*(0) = 1.2. Note that if *τ* ≥ 17, the time series shows a chaotic behaviour [33, 34]. The nominal Mackey-Glass time series is obtained from numerical integration by a fourth order Runge-Kutta method. This series was computed with a time sampling of 1 second. Thus, *x*(*t*) is derived from 0 ≤ *t* ≤ *t*_*h*_ with *x*(*t*) = 0 for *t* < 0, where *t*_*h*_ is the time horizon considered.

Mackey-Glass chaotic time series with *τ* = 17 is considered as the nominal case *x* ^Noiseless^ (without noise contribution). Here, we generate two thousand data points (*t*_*h*_ = 2000).

From this data set, the input is created as a vector using *d* points of the time series spaced Δ apart; that is, **x**(*t*) = [*x*(*t*), *x*(*t* + Δ),…, *x*(*t* + (*d* − 1)Δ)]. The output is generated with the value *x*(*t* + *T*).

According to the standard analysis of the Mackey-Glass chaotic time series, we consider four nonconsecutive points in the chaotic time series in order to predict the short-term *x*(*t* + 6):

(13) $$\begin{array}{c} x\left(t+6\right)=F\left[x\left(t\right),x\left(t-6\right),x\left(t-12\right),x\left(t-18\right)\right], \end{array}$$

where this standard test assumes *d* = 4 and Δ = *T* = 6 [6, 34].

For this input, the first thousand data sets were used for learning (*training*), while the others were used for the prediction validation (*prediction*). In the ANN+PSO implementation on the nominal case, the optimum value of *N*_HL_ found was six; that is, the architecture is described as 4-6-1.

**Figure 2:**
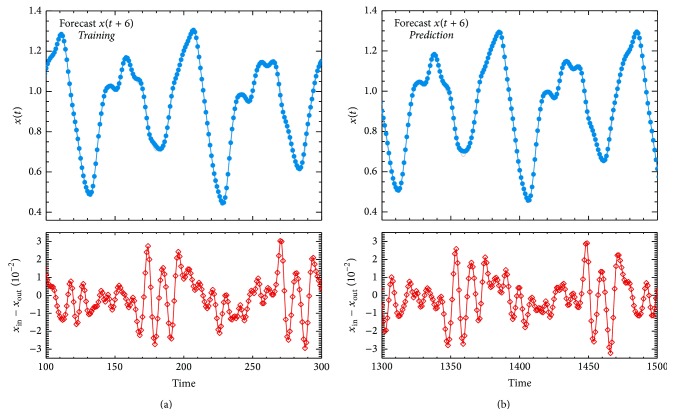
Performance of ANN+PSO method on the Mackey-Glass chaotic time series (noiseless). (a) and (b) show the* training* and* prediction* performance for the short-term *x*(*t* + 6) analysis, respectively. The grey and blue lines correspond to the input (*x*_in_) and output (*x*_out_) data. The red line with diamond draws the difference between the input and output data (in a factor of 10^−2^).

Figure 2 presents a comparison between recorded and predicted values of the Mackey-Glass time series for the training and prediction phases. This figure shows that, for training and validation phases, the nominal and reconstructed values are in total agreement. In fact, for training, we computed a remainder average, 〈*x*_in_ − *x*_out_〉, of  −1.4 × 10^−3^ and a remainder maximum, max⁡{|*x*_in_ − *x*_out_|}, of 3.20 × 10^−2^. Similar results are obtained for the prediction phase, with a maximum of 3.22 × 10^−2^ and an average of  −1.5 × 10^−3^.

Table 2 shows the RMSE (for short-term prediction of Mackey-Glass chaotic time series) from different computational methods obtained from literature, for example, the backpropagation NN [35], the conjugate gradient ANN [36], the product operator *T*-norm [37], and the fuzzy system [38] (see references in Table 2). In the ANN+PSO configuration used here, the RMSE = 0.014 indicates that the performance prediction is in good agreement with other methods. Clearly, the inclusion of the PSO approach allows us to improve methods based on ANN without PSO, for example, the conjugate gradient ANN (RMSE = 0.229) and the backpropagation NN (RMSE = 0.026).

### 3.1. Chaotic Behaviour

**Figure 3:**
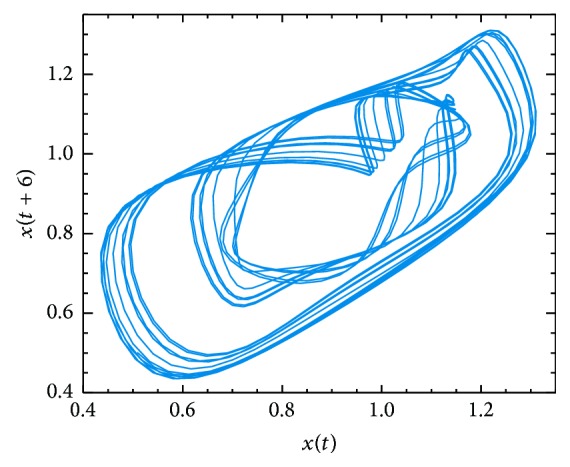
Chaotic attractor for the Mackey-Glass noiseless chaotic time series (*τ* = 17).

As the Mackey-Glass time series without noise is a known system, it is possible to compare the ability of ANN+PSO method of reproducing its chaotic behavior.  Figure 3 shows a representation of the chaotic attractor studied from Mackey-Glass time series. This figure shows that with *τ* = 17 the system operates in a high-dimensional regime. The Mackey-Glass system is infinite dimensional system (because it is a time-delay equation) and, thus, has an infinite number of Lyapunov exponents (*λ*_*i*_) [33]. The Lyapunov exponents of dynamical systems are one of a number of invariants that characterize the attractors of the system in a fundamental way [43].  Table 3 shows a comparison of the first four largest Lyapunov exponents of the Mackey-Glass system reported in [33], with the Lyapunov exponents obtained for the ANN+PSO method for *τ* = 17.

An approach to determine an appropriate cutoff value for the number of exponents can be related to the Lyapunov dimension [43]. This idea was originally explored by Kaplan and York [44]. Thus, Kaplan and York conjecture that this dimension (*D*_KY_) is equal to the information dimension [45]. In our case, *D*_KY_ is computed as 2.10. Note that, in Farmer [33], the authors reported a fractal dimension *D*_F_ = 2.13 and a Lyapunov dimension calculated by the Kaplan-York conjecture of *D*_KY_ = 2.10.

## 4. Noisy Chaotic Time Series Prediction

In the previous section, the ANN+PSO has proven to be an efficient method to the prediction of chaotic time series. Nevertheless, up to now, effects of noise on the hybrid ANN+PSO implementation have not been studied.

In order to study the impact of noise on chaotic series time prediction, we constructed the noisy time series as the contribution of a noise level on the nominal case without noise. The Mackey-Glass noisy chaotic time series, *x*_*i*_ ≡ *x*(*t*), is generated as

(14) $$\begin{array}{c} x_{i}=x_{i}^{Noiseless}+\eta_{i}, \end{array}$$

where *η*_*i*_ is the particular contribution of noise on the *i*-element. It is estimated as *η*_*i*_ = GR(*σ*_*N*,*i*_), with GR(*σ*_*N*,*i*_), a Gaussian random number generator.

**Figure 4:**
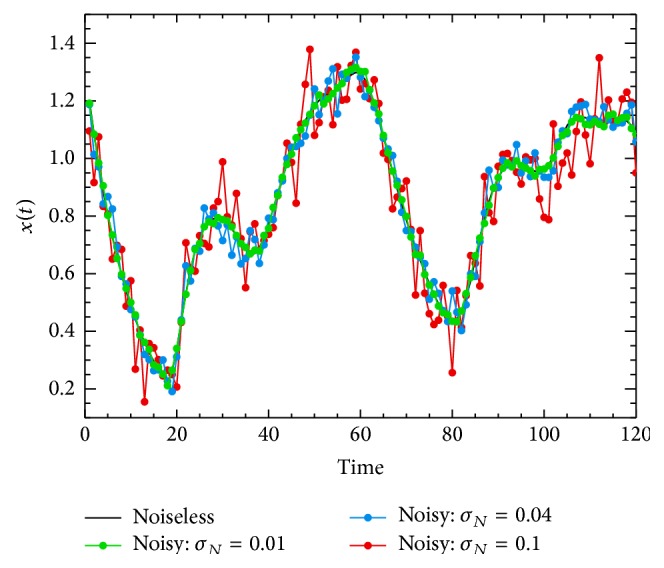
Mackey-Glass chaotic time series considered in this work (*τ* = 17). The black solid line shows the noiseless case (nominal case). The green, blue, and red lines correspond to the Mackey-Glass noisy time series with a white noise level (*σ*_*N*_) contribution of 0.01, 0.04, and 0.1, respectively.

Note that *σ*_*N*,*i*_^2^ corresponds to the noise level considered. Here, we assume that the original data are effected by a white noise; that is, the noise level is the same in each *i*-element, *σ*_*N*,*i*_ = *σ*_*N*_ (for clarification, although the noise level *σ*_*N*_ is the same in each time, the noise contribution *η*_*i*_ is not the same (the latter depends on the Gaussian random number generator)). Different white noise levels are considered: *σ*_*N*_ = 0.01, *σ*_*N*_ = 0.04, *σ*_*N*_ = 0.06, *σ*_*N*_ = 0.08, and *σ*_*N*_ = 0.1. These values are nearly related to the 1%, 4%, 6%, 9%, and 11% of the pick-to-pick amplitude of nominal case (~*x*_max_^Noiseless^ − *x*_min_^Noiseless^).  Figure 4 shows that the noisy chaotic time series for *σ*_*N*_ is equal to 0.01 (green), 0.04 (blue), and 0.1 (red). As expected, the noisy time series with *σ*_*N*_ = 0.01 is the closest to the nominal case. However, the cases with *σ*_*N*_ = 0.04 and *σ*_*N*_ = 0.1 show a slightly more modified shape from the noiseless case, in particular with *σ*_*N*_ = 0.1.

### 4.1. Noise Effect on ANN+PSO

The* standard* ANN+PSO is applied to our noisy time series, which provides the optimum topology and the *y*_*i*_ prediction. Then, the* stochastic* ANN+PSO is run in order to obtain a new prediction estimator ${\hat{y}}_{i}$ and the uncertainty of the prediction ($\sigma_{{\hat{y}}_{i}}$).

**Figure 5:**
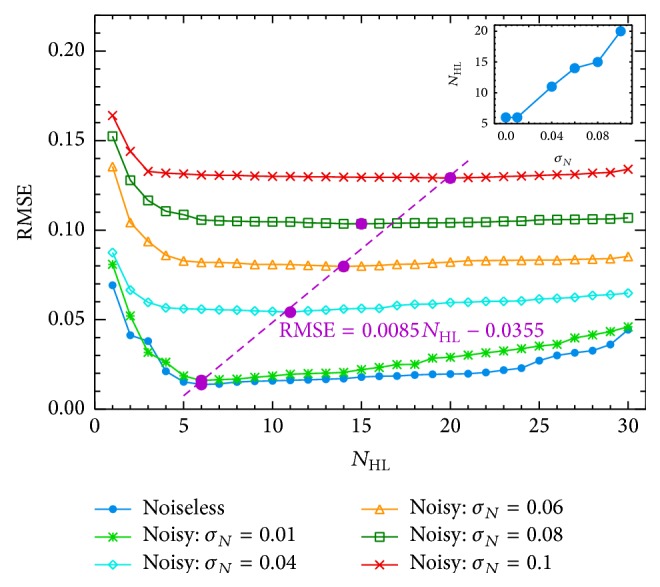
Impact of the noise on the architecture.

*Impact on Architecture.* For each noisy time series, in the* standard* ANN+PSO implementation, we carry out a detailed study of the architecture characterization. In the determination of the optimum *N*_HL_, the RMSE is computed for different number of neurons in the hidden layer (from two up to thirty), which are presented in Figure 5. For each series, the optimum *N*_HL_ is obtained when the RMSE reaches a minimum. As expected, the characterization of the architecture is strongly related to the noise level in the input data. In lower noise (as 0.01), the optimum *N*_HL_ is clearly identified from Figure 5; in contrast, in the most contaminated case (*σ*_*N*_ = 0.1), the selection depends on the fourth decimal of the RMSE (0.1292, 0.1291, and 0.1293 for 19, 20, and 21 neurons in the hidden layer, resp.). The RMSE and the *N*_HL_ optimum are presented in Table 4. Using these values and according to the trend seen in Figure 5, we fit a lineal model, which provides a correlation with a slope of 0.0085. Although the *N*_HL_ for *σ*_*N*_ = 0.08 is not well characterized for this model, we can find a clear lineal correlation between the RMSE and the *N*_HL_ for different noise levels. In this context, as an illustration, in the overplot (in top-right side of Figure 5), we show the relation of the *N*_HL_ and the noise level, whose best lineal fit model is *N*_HL_ = 146*σ*_*N*_ + 4.7. Therefore, the impact of noise on the architecture of this hybrid neural network, for contributions lower than 0.1, can be characterized by a lineal correlation of the RMSE with the *N*_HL_ and the *N*_HL_ with the input noise *σ*_*N*_.

**Figure 6:**
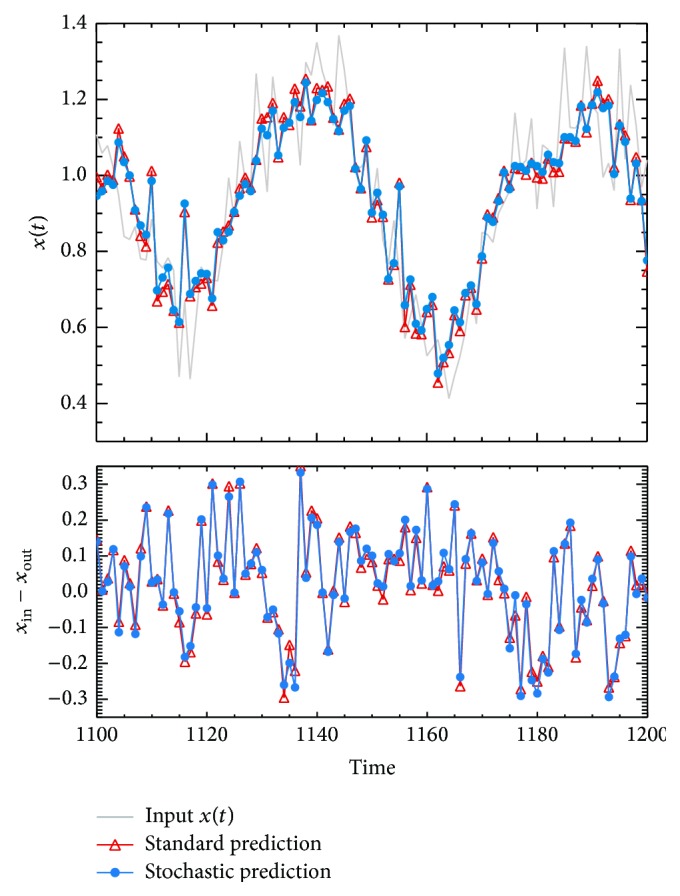
Predictions of Mackey-Glass noisy chaotic time series with a white noise contribution of *σ*_*N*_ = 0.1. The grey solid line corresponds to the original Mackey-Glass noisy chaotic time series. The red and blue lines identified the results from* standard* ANN+PSO and* stochastic* ANN+PSO, respectively. The upper panel draws the *y*_*i*_ and ${\hat{y}}_{i}$ predictions, and the lowe panel draws the residual contribution (*x*_in_ − *x*_out_) of both methods.

*The Prediction Performance.* As an illustration, the predictions obtained for noisy case *σ*_*N*_ = 0.1, from the* standard* ANN+PSO (*y*_*i*_) and the* stochastic* ANN+PSO (${\hat{y}}_{i}$) procedures, are presented in Figure 6. As expected, even on this high noise level case, the *y*_*i*_ and ${\hat{y}}_{i}$ predictions are in total agreement. Actually, the RMSE obtained from both methods is the same (in the approximation of the third decimal) for each noisy case. For this reason, the RMSE shown in Table 4 represents the RMSE of both methods.

**Figure 7:**
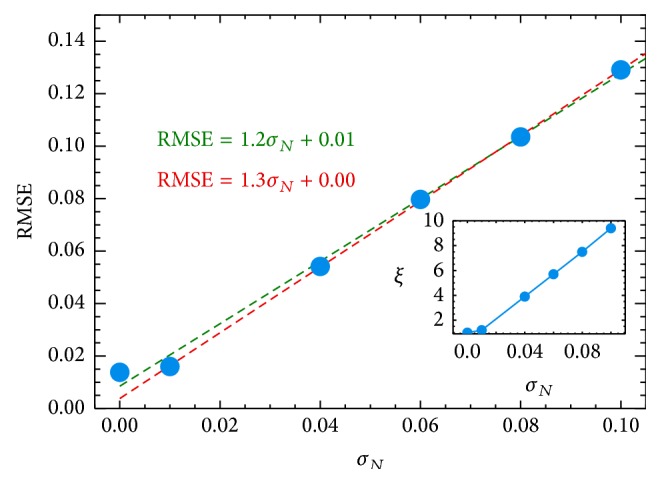
Impact of the noise on the performance prediction.

On the other hand, as expected, the RMSE increases with the growing the noise level (see Figure 7). For example, we obtained RMSE of 0.0138 and 0.13 for the noiseless and noisy (with *σ*_*N*_ = 0.1) cases, respectively. From Figure 7, we observe a linear correlation between the RMSE and the input noise level. The best fit model, without considering the RMSE of the noiseless case, corresponds to RMSE = 1.3*σ*_*N*_, which shows a strong lineal correlation. Therefore, we confirm that a higher noise level in input data leads to a poor estimation of the prediction estimator, which is related linearly to the input noise level.

Also, the ratio *ξ* = RMSE_noisy_/RMSE_noiseless_ (third column in Table 4) can be used to study the impact of noise on the performance efficiency of our implementation (with respect to nominal case). The bottom-right panel of Figure 7 shows the performance efficiency against the input noise level. In the worst case, the performance efficiency (*ξ*) is strongly affected by one order of magnitude with respect to the noiseless case. Even so, the* standard* and* stochastic* ANN+PSO confirm to be a powerful tool for making predictions of chaotic time series.

In the literature, we do not find a similar implementation (due to the ahead prediction, type and level of noise, etc.) that allows for us a straightforward comparison of results. For example, we can contrast our results with those presented by Sheng et al. 2012 [14]. They applied the Echo State Network (ESN) based on dual estimation on a noisy Mackey-Glass time series (with a sampling of 2 seconds) with a white noise level of *σ* = 0.1. However, the prediction ahead was one, which is considered lower than ours. Yet, let us carry out a plain comparison. Depending on the prediction performance, they obtained RMSE of 0.05 for Generic ESN (hereafter GESN) and 0.04 for CKF/KF based ESN (henceforth CESN). In this context, the impact of the noise on the performance efficiency is lower in ANN+PSO implementation (with respect to the ESN). In fact, we have a performance efficiency *ξ* of 9.4, while they obtained *ξ* of 1161 and 33.5 for GESN and CESN, respectively.

**Figure 8:**
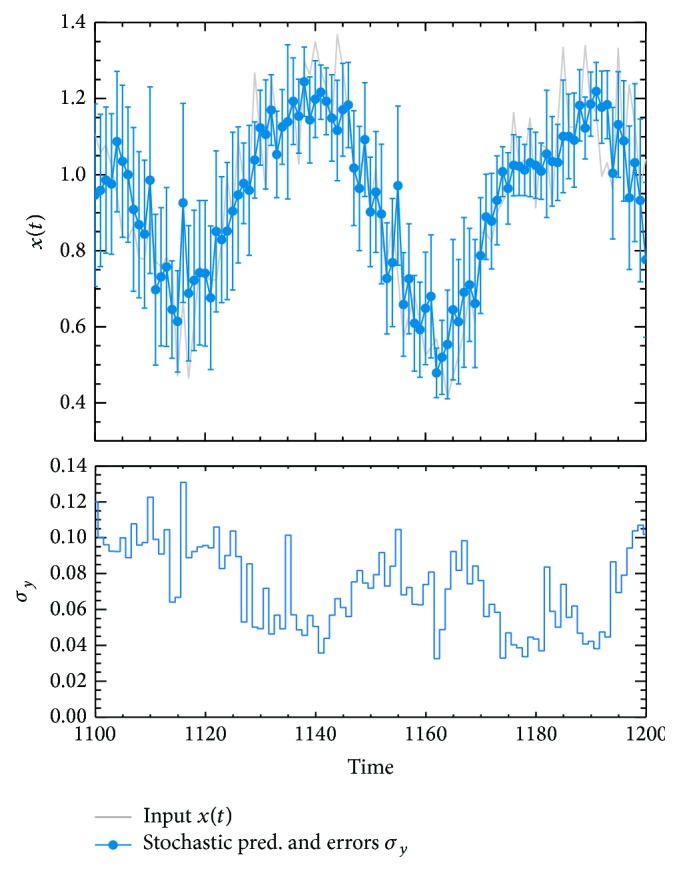
Predictions and uncertainties from the* stochastic* ANN+PSO for the Mackey-Glass chaotic time series. This corresponds to the case with a white noise of *σ*_*N*_ = 0.1. In the upper panel, the gray solid line draws the original Mackey-Glass noisy chaotic time series. The blue points with error bars correspond to the ${\hat{y}}_{i}$ prediction and their uncertainties $\sigma_{\hat{y}}$. For optimal display of the uncertainties, these are presented in the lower panel.

*Prediction Uncertainties.* One of the main goals of this work is to estimate the uncertainty on the prediction. The prediction measurement (${\hat{y}}_{i}$) and the error bars ($\sigma_{{\hat{y}}_{i}}$) obtained from the* stochastic* ANN+PSO, for the noisy time series with *σ*_*N*_ = 0.1, are presented in Figure 8. We confirm that our forecast and input data, for the strong noise contribution, are in agreement at one sigma (at 68.5% of confidential level) when the error bars are considered. The uncertainties obtained are presented in the low panel of Figure 8. We found a minimum and maximum uncertainty of 0.024 and 0.13, respectively, with an average of $\langle \sigma_{{\hat{y}}_{i}}\rangle =0.07$. This value is lower than the input noise level ($\langle \sigma_{{\hat{y}}_{i}}\rangle /\sigma_{N}=0.7$), and this shows the impact of the error propagation in our methods. According to Figure 8, a relationship between the uncertainties and the times is not appreciated.

Finally, from Figures 6 and 8, we have proven that ANN+PSO (with the* standard* and/or the* stochastic* implementation) is a robust tool in the predictability (for the short-term prediction) of time series affected by a white noise. In addition, now the ANN+PSO method can provide, for first time, an estimation of the uncertainty of the prediction.

## 5. Conclusions

In this paper, a hybrid algorithm based on artificial neural network and particle swarm optimization (ANN+PSO) is used in the short-term *x*(*t* + 6) prediction of Mackey-Glass chaotic time series. In addition, a study of the impact of the noise on our hybrid method is presented. Based on the results and discussion presented in this study, we have the following conclusions.

(i) The current value *x*(*t*) and the past values used have influential effects on the good training and predicting capabilities of the chosen network.
(ii) In noiseless case, simulation shows that this hybrid ANN+PSO algorithm is a very powerful tool for making prediction of chaotic time series, and the low deviations found with the proposed method show an accuracy comparable with other methods available in the literature.
(iii) In noisy cases, we have proven that the hybrid ANN+PSO is a robust tool in the predictability of the short-term prediction of chaotic time series affected by a white noise.
(iv) The impact of the noise on the topology and performance efficient of the ANN+PSO is important. However, this study shows that the error propagation through the ANN+PSO has a linear behaviour, which generates a linear relationship between the RMSE (optimization parameter) and the input noise level. Therefore, the PSO optimization provides a linearity which ensures that the neural network will converge to an appropriate solution, even if a noise level contribution is present.
(v) For noisy cases, although a straightforward comparison with literature is unavailable, the performance efficient *ξ* proves that the* standard*/*stochastic* ANN+PSO implementation is affected in a lesser degree than the other similar performances.

## Figures and Tables

**Table 1:** Parameters used in the hybrid ANN+PSO algorithm.

ANN	
NN-type	Feed-forward
Number of hidden layers	1
Transfer function	*Tansig *
Number of iterations	1500
Normalization range	[−1,1]
Weight range	[−100,100]
Bias range	[−10,10]
Minimum error	1*e* − 3

PSO	

Number of particles in swarm (*N*_part_)	50
Number of iterations (*k*_max⁡_)	1500
Cognitive component (*c*_1_)	1.494
Social component (*c*_2_)	1.494
Maximum velocity (*v*_max⁡_)	12
Minimum inertia weight (*ω*_min⁡_)	0.5
Maximum inertia weight (*ω*_max⁡_)	0.7
Objective function	RMSE

**Table 2:** Root mean squared error (RMSE) reported for different methods in the Mackey-Glass chaotic time series analysis.

Method	RMSE_*x*(*t*+6)_
Linear model [35]	0.5503
Conjugate gradient ANN [36]	0.2296
Product operator *T*-norm [37]	0.0907
Fuzzy system [38]	0.0816
Cascade correlation NN [39]	0.0624
Genetic algorithm and fuzzy system [40]	0.0490
Backpropagation NN [35]	0.0262
Linguistic model (20 rules) [41]	0.0256
*K*-nearest neighbor [42]	0.0194
This work	0.0138

**Table 3:** Lyapunov exponents reported in Farmer [33] versus those calculated for the ANN+PSO method.

*λ*_*i*_	*λ*_*i*,ANN+PSO_
0.00860	0.00900
0.00100	0.00132
−0.03950	−0.04100
−0.05050	−0.05000

**Table 4:** Parameters used in the evaluation of the prediction performance of the *standard* and *stochastic* ANN+PSO approach.

	*N*_HL_	RMSE	*ξ*
Noiseless	6	0.0138	1
*σ*_*N*_ = 0.01	6	0.016	1.2
*σ*_*N*_ = 0.04	11	0.054	3.9
*σ*_*N*_ = 0.06	14	0.078	5.7
*σ*_*N*_ = 0.08	15	0.103	7.5
*σ*_*N*_ = 0.1	20	0.129	9.4
